# Thermal Rheology and Fibrous Structure of High-Moisture Meat Analogs with Hemp Seed Cake

**DOI:** 10.3390/gels12090773

**Published:** 2026-08-28

**Authors:** Hyerim Jeon, Bon-Jae Gu

**Affiliations:** Department of Food Science and Technology, Food and Feed Extrusion Research Center, Kongju National University, Yesan 32439, Republic of Korea

**Keywords:** hemp seed cake, high-moisture extrusion, meat analog, thermal rheology, fibrous structure, food upcycling

## Abstract

The utilization of protein-rich agricultural by-products offers a sustainable strategy for developing plant-based meat analogs. This study investigated the effects of cold-pressed hemp seed cake (HSC) incorporation at levels of 0–20% on the pasting, thermal–rheological, textural, and structural properties of high-moisture meat analogs produced by extrusion. Increasing HSC incorporation significantly reduced peak viscosity, indicating altered starch–protein–fiber interactions within the blends. During temperature-sweep measurements, all formulations exhibited elastic-dominant behavior, with the storage modulus remaining higher than the loss modulus throughout heating and cooling. Although the initial viscoelastic moduli decreased with increasing HSC content, the final moduli after cooling were comparable to those of the control. Incorporation of 15% and 20% HSC significantly decreased hardness from 44.02 to 38.88 N and chewiness from 1730.20 to 1517.31 g, whereas springiness, cohesiveness, and the hardness degradation ratio remained largely unchanged. Fibrous structures were maintained in all formulations, while cutting strength tended to increase and the texturization degree numerically increased from 1.04 to 1.15 at 20% HSC. These findings demonstrate that HSC can replace up to 20% of the conventional protein–starch blend while maintaining thermal viscoelasticity and anisotropic fibrous structure, although producing a moderately softer high-moisture meat analog.

## 1. Introduction

The growing demand for dietary protein and environmental concerns associated with conventional livestock production have intensified the need for sustainable food alternatives [1]. Consequently, plant-based meat analogs (PBMA) have gained considerable attention due to their potential to reduce environmental burdens [2]. While plant proteins provide valuable nutrients and amino acids, their techno-functional and nutritional properties vary depending on the protein source, product formulation, and processing method [3].

Hemp seed has emerged as a promising functional food ingredient because of its relatively high protein content, generally ranging from 20% to 25%, as well as its unsaturated fatty acids, dietary fiber, and bioactive constituents [4,5]. Hemp proteins exhibit favorable digestibility and contain indispensable amino acids, including leucine, lysine, and phenylalanine [6]. Owing to these compositional and nutritional characteristics, interest in hemp-based food ingredients and products has increased in recent years [7]. Hemp seeds are commonly processed for oil production, particularly through cold pressing, which generates hemp seed cake (HSC) as a major by-product. Although HSC remains rich in protein, dietary fiber, and residual lipids, its utilization in food processing remains relatively limited. Edestin, the predominant storage protein in hemp seed, is a globular protein that shares structural characteristics with other plant storage globulins, including soy globulins [8]. These protein characteristics suggest that HSC may contribute to intermolecular interactions and structural organization in mixed protein systems. In addition, hemp seed components and oil-processing by-products have been proposed as partial substitutes for conventional ingredients derived from palm oil, soy, and wheat in food formulations, thereby supporting more sustainable use of agricultural resources [9]. Nevertheless, HSC differs considerably from refined protein ingredients because it contains not only proteins but also dietary fiber, residual lipids, and other non-protein constituents. These components may affect water distribution, starch gelatinization, protein hydration, melt flow, and network formation during food processing. Therefore, the functionality of HSC cannot be predicted solely from its protein content, and its behavior under thermomechanical processing conditions requires systematic evaluation.

High-moisture extrusion (HME) is widely used to produce meat analogs with fibrous and anisotropic structures through the combined effects of heat, shear, pressure, moisture, and cooling [10,11]. Compared with conventional low-moisture extrusion, HME uses a higher water content, which increases molecular mobility and facilitates phase separation and structural rearrangement within the protein-rich melt [12]. Under these conditions, proteins undergo conformational changes and intermolecular associations, while the cooling die promotes alignment and stabilization of the multiphase structure. These processes contribute to the development of the characteristic fibrous architecture of high-moisture meat analogs [13]. The incorporation of HSC into an HME formulation may influence these structural transitions through several competing effects. Hemp proteins may participate in the development of the mixed protein network, whereas dietary fiber may alter water availability and interfere with protein hydration or continuous matrix formation. Residual lipids may additionally modify lubrication, shear transfer, and the mobility of components within the extrusion melt. Consequently, the final structure of HSC-containing meat analogs is likely to depend on the combined effects of its protein, fiber, and lipid fractions rather than on a single component. Thermal–rheological measurements can provide useful information regarding these effects because changes in storage modulus and loss modulus during heating and cooling reflect the evolution of elastic and viscous characteristics within the formulation. These measurements can help assess how HSC affects thermally induced gel-like network development and its relationship with the texture and fibrous structure of the final extrudates.

While previous studies have demonstrated the feasibility of utilizing purified hemp protein concentrates or isolates in HME [14,15], research on directly incorporating whole, cold-pressed HSC into plant-based meat matrices remains scarce. Utilizing unrefined HSC not only promotes circular bioeconomy and food upcycling, but also provides complex functional contributions from naturally co-existing proteins, fiber, and lipids that differ markedly from commercial isolates. Its simultaneous contributions of proteins, dietary fiber, and residual lipids may produce different effects on pasting behavior, thermal viscoelasticity, texture, and fibrous structure. Nevertheless, the relationships among HSC incorporation, thermal–rheological behavior, and the quality characteristics of high-moisture meat analogs remain insufficiently understood.

Therefore, this study investigated the effects of incorporating 0–20% cold-pressed HSC into a soy protein isolate–wheat gluten–corn starch formulation used to produce high-moisture meat analogs. The pasting and thermal–rheological properties of the formulations were evaluated together with the appearance, expansion ratio, bulk density, color, texture, cutting strength, and texturization degree of the extruded products. The study aimed to determine how partial replacement of conventional protein–starch ingredients with HSC affects thermally induced viscoelastic network development and the formation of anisotropic fibrous structures. The findings provide information regarding the potential utilization of HSC as an upcycled ingredient in high-moisture extrusion systems and its influence on the physicochemical and structural properties of plant-based meat analogs.

## 2. Results and Discussion

### 2.1. Effects of HSC Content on Pasting Properties

The pasting properties of the HMMA blends containing different levels of HSC powder are presented in Table 1. Peak viscosity (PV) decreased significantly as the HSC content increased (*p* < 0.05), declining from 2098.33 ± 97.50 cP in the control to 1257.67 ± 17.01 cP in the formulation containing 20% HSC. This continuous reduction indicates that HSC incorporation substantially limited the maximum viscosity development of the blends during heating. Trough viscosity (TV), breakdown viscosity (BV), final viscosity (FV), and setback viscosity (SB) also differed significantly among the formulations (*p* < 0.05). However, unlike PV, these parameters did not show consistent dose-dependent changes across the entire HSC concentration range. TV decreased markedly following the incorporation of 5% HSC and subsequently remained at relatively similar levels, whereas BV increased at 5% and 10% HSC before decreasing at the higher incorporation levels. FV and SB generally showed lower values at 15% and 20% HSC than in the control, indicating that higher HSC incorporation affected viscosity development during both heating and subsequent cooling. In addition, while pasting temperature (PT) remained constant at 50.00 °C across all formulations (*p* > 0.05), peak time increased progressively from 7.64 ± 0.08 min in the control to 11.64 ± 0.20 min at 20% HSC addition (*p* < 0.05).

The observed changes in pasting behavior may be associated with the compositional characteristics of HSC. Cold-pressed HSC contains substantial quantities of protein and dietary fiber together with residual lipids [16]. Because HSC proportionally replaced isolated soy protein, wheat gluten, and corn starch in the formulation, increasing its incorporation reduced the relative starch content while introducing additional non-starch components. The lower proportion of starch likely contributed to the progressive reduction in PV because less starch was available to swell and contribute to viscosity development during heating. In addition, the protein, dietary fiber, and residual lipids present in HSC may have competed with starch for available water or physically restricted starch granule swelling. Previous studies have demonstrated that incorporating protein- and fiber-rich plant materials into starch-based systems can alter pasting properties by changing water availability, starch hydration, and interactions within the continuous matrix [17,18].

Interactions among starch, proteins, and lipids may also have contributed to the reductions in FV and SB observed at higher HSC levels. In model starch systems, proteins and free fatty acids have been reported to suppress viscosity development during heating and cooling through multicomponent interactions that limit starch swelling and reassociation [19]. Accordingly, the lower FV and SB values of the 15% and 20% HSC formulations may indicate reduced development of the cooled paste structure. The marked decrease in BV at these levels may similarly reflect reduced structural breakdown during heating; however, the non-linear response at 5% and 10% HSC suggests that the pasting behavior was governed by complex interactions rather than by HSC concentration alone.

Overall, HSC incorporation modified the pasting profile of the HMMA blends, with the most consistent effect being the progressive reduction in PV. These changes suggest that HSC altered the hydration and viscosity development of the protein–starch matrix before extrusion. The reductions in PV, FV, and SB provide useful evidence that HSC incorporation changed the pre-extrusion functionality of the blends and may consequently influence their flow behavior and structural development during HME. In particular, the prolonged peak time further reflects that insoluble dietary fiber and hydrophobic lipid constituents in HSC delayed water diffusion into the starch granules, thereby retarding starch gelatinization and the attainment of PV.

### 2.2. Effects of HSC Content on Thermal–Rheological Properties

**Figure 1:**
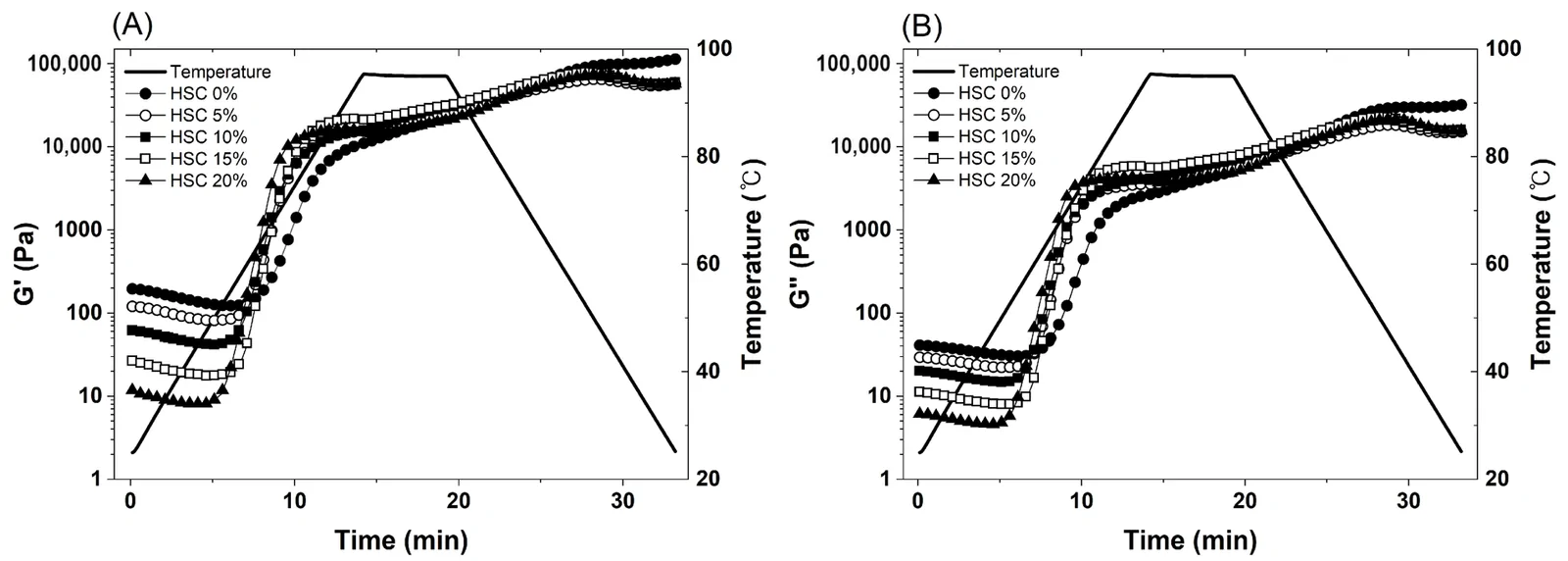
Thermal–rheological properties of HMMA blends containing different levels of HSC powder: (**A**) storage modulus (G′) and (**B**) loss modulus (G″).

The thermal–rheological properties of the formulations were evaluated under dynamic temperature conditions to characterize changes in their viscoelastic behavior during heating and cooling. These measurements provide information on the development and reinforcement of gel-like structures in the HMMA blends. The thermal–rheological profiles of the blends, represented by the storage modulus (G′) and loss modulus (G″), are shown in Figure 1A,B, respectively. Throughout the entire temperature cycle, G′ remained higher than G″ in all formulations, indicating predominantly elastic and gel-like behavior. The absence of a crossover between G′ and G″ further suggests that the blends maintained an elastic-dominant structure during both heating and cooling.

In the initial temperature range of 25–50 °C, both G′ and G″ tended to decrease with increasing HSC content. This reduction indicates that HSC incorporation weakened the initial viscoelastic structure of the hydrated blends before substantial thermal transitions occurred. Because HSC proportionally replaced isolated soy protein, wheat gluten, and corn starch, increasing the HSC content altered the relative proportions and functional characteristics of the matrix components. Compared with the refined ingredients, HSC contains a more heterogeneous mixture of proteins, dietary fiber, and residual lipids [16]. Its incorporation may therefore have changed water distribution, particle hydration, and intermolecular associations within the blends. The residual lipids in HSC may also have exerted a lubricating or plasticizing effect, thereby reducing friction and interactions among hydrated particles.

During heating, both G′ and G″ increased rapidly in all formulations, particularly within the temperature range of approximately 80–90 °C. The marked increase in the viscoelastic moduli indicates the development of a thermally induced network within the blends. This transition may have resulted from the combined effects of protein conformational changes, increased protein–protein associations, and starch gelatinization. Because the formulations contained isolated soy protein, wheat gluten, corn starch, and HSC, the observed modulus increase likely represented overlapping thermal transitions rather than the behavior of a single component. Importantly, all HSC-containing formulations exhibited a similar upward transition during heating, indicating that incorporation of up to 20% HSC did not prevent the development of an elastic-dominant structure. During cooling from 95 to 25 °C, both G′ and G″ increased further across all formulations and reached their highest values at the end of the temperature cycle. This increase indicates progressive reinforcement of the viscoelastic network as the temperature decreased. This cooling-induced strengthening can likely be attributed to the stabilization and physical compaction of the blended network as thermal energy decreases, accompanied by reduced molecular mobility and progressive matrix reassociation during cooling.

Despite the lower initial G′ and G″ values observed at higher HSC levels, the final moduli of the HSC-containing blends were comparable to those of the control formulation. This result indicates that HSC incorporation weakened the initial hydrated structure but did not substantially reduce the ability of the blends to develop a reinforced gel-like network during heating and cooling. Hemp seed proteins, particularly edestin and albumin, possess globular structures and functional groups that may participate in intermolecular associations with isolated soy protein and wheat gluten [8,20,21]. These proteins may therefore have contributed to the recovery and reinforcement of the viscoelastic structure during the thermal cycle. Similar increases in G′ and G″ during heating and cooling have been reported in plant protein gel systems [22]. In the present study, the thermal–rheological results demonstrate that HSC modified the initial viscoelastic properties of the formulations while preserving their ability to form an elastic-dominant and reinforced gel-like structure after heating and cooling. This retained thermal-network-forming capacity may help explain why the macroscopic fibrous structure of the extruded products was maintained even at the highest HSC incorporation level.

### 2.3. Appearance of HMMA

**Figure 2:**
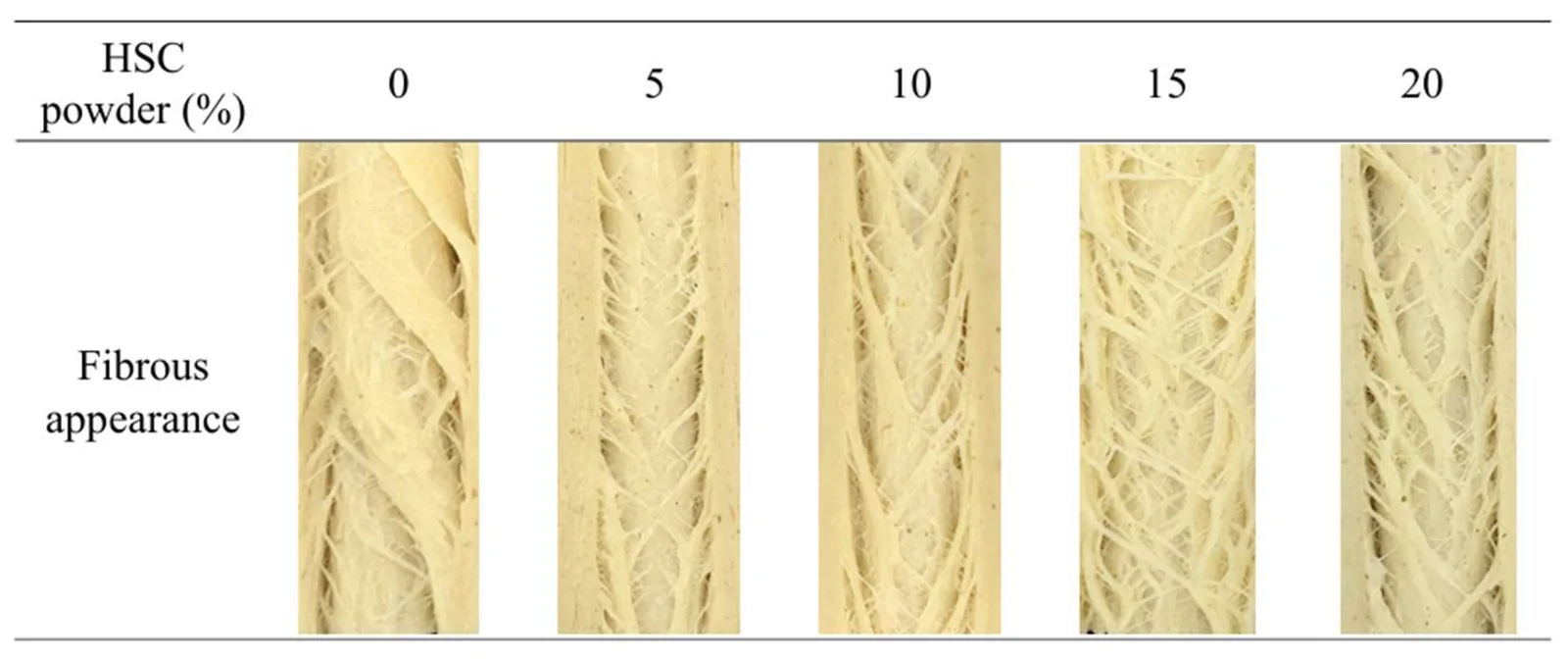
Photographs of the appearance with fibrous structure of HMMA at different levels of HSC powder content (samples were incised longitudinally and pulled apart to expose the internal fibrous structure).

The appearances of the HMMA containing different levels of HSC powder are shown in Figure 2. All extrudates exhibited visible fibrous structures, and no marked deterioration of the overall macrostructure was observed as the HSC content increased from 0% to 20%. The control formulation displayed an oriented fibrous appearance typical of HMMA, while the HSC-containing formulations maintained comparable macroscopic fiber formation. At higher HSC levels, particularly 15% and 20%, the extrudates appeared to exhibit relatively compact fibrous regions. The incorporation of fiber-rich ingredients into HME formulations can affect protein–starch matrix development and may weaken structural continuity under certain processing conditions [23]. Dietary fiber has a high affinity for water and may compete with proteins and starch for available moisture, thereby influencing protein hydration and thermal transitions [24]. Reduced water availability may also alter starch gelatinization and the formation of a continuous matrix during extrusion [25].

Despite the dietary fiber present in HSC, no clear structural deterioration was observed in the HSC-containing extrudates in the present study [26]. This result may be related to the combined contributions of proteins, dietary fiber, and residual lipids in HSC. Hemp seed proteins, including edestin, may participate in intermolecular interactions within the mixed protein matrix [20]. Residual lipids may also affect melt flow, lubrication, and structural organization during extrusion. Previous research reported that oil incorporation altered protein interactions and texturization in high-moisture extruded protein systems [27]. Consistent with the visual observations, the texturization degree showed a numerical increase from 1.04 in the control to 1.15 at 20% HSC. Therefore, HSC incorporation up to 20% did not impair the formation of the macroscopic fibrous structure under the extrusion conditions applied in this study.

### 2.4. Expansion Ratio (ER) and Bulk Density (BD)

As shown in Table 2, the expansion ratio and bulk density of the HMMA formulations were evaluated to determine the effects of HSC incorporation on the physical characteristics of the extrudates. Expansion ratio and bulk density generally exhibit an inverse relationship, as an increase in extrudate volume relative to mass commonly results in a lower apparent density. Although neither parameter showed a consistent dose-dependent trend with increasing HSC content, the formulation containing 20% HSC exhibited the highest expansion ratio and the lowest bulk density. Specifically, the expansion ratio increased from 1.17 in the control to 1.24 at 20% HSC, whereas bulk density decreased from 0.99 to 0.91 g/cm^3^ (*p* < 0.05). These results indicate that HSC incorporation up to 15% produced relatively limited changes in overall expansion behavior, while the 20% formulation resulted in a more expanded and less dense extrudate structure.

The expansion behavior of high-moisture extrudates is influenced by complex interactions among moisture, proteins, starch, dietary fiber, and lipids within the extrusion matrix. Previous research has suggested that the structural effects of fiber-rich ingredients under HME conditions may be moderated by the formation of a sufficiently cohesive protein matrix [28]. Although dietary fiber can compete for available water and interfere with continuous matrix development, its effects may vary according to raw material composition and the extent of protein hydration and aggregation. In the present study, the proteins and residual lipids supplied by HSC may have modified melt flow and matrix organization, thereby offsetting the potentially disruptive effects of its dietary fiber fraction. The relatively high moisture content used during extrusion may also have supported component mobility and structural rearrangement within the matrix. These combined effects may partially explain why the 20% HSC formulation showed a higher expansion ratio and lower bulk density while maintaining the overall structural integrity of the extrudate.

### 2.5. Color Properties

The color properties of the HMMA containing different levels of HSC powder are presented in Table 2. Color is an important quality attribute of plant-based meat analogs because it can influence consumer perception and product acceptability [29,30]. Accordingly, the CIE color coordinates, including lightness (L*), redness (a*), and yellowness (b*), together with the total color difference (ΔE), were evaluated to determine how HSC incorporation affected the visual characteristics of the HMMA [31].

The L* values did not exhibit a consistent dose-dependent trend with increasing HSC content. The control formulation showed an L* value of 76.52, whereas the 10% HSC formulation exhibited the highest value of 77.27 (*p* < 0.05). In contrast, the 5% and 20% HSC formulations showed lower L* values of 74.92 and 74.90, respectively (*p* < 0.05). Although significant differences were observed among the formulations, the overall numerical range of L* values was relatively narrow. This result indicates that HSC incorporation produced only moderate changes in product lightness rather than a continuous darkening or lightening effect. The a* values also changed with HSC incorporation. The control and 5% HSC formulations showed relatively similar a* values of 2.59 and 2.68, respectively (*p* < 0.05), whereas the values decreased to 2.08, 1.99, and 2.06 at 10%, 15%, and 20% HSC, respectively (*p* < 0.05). These results indicate that higher HSC incorporation reduced the redness of the HMMA. In contrast, the b* values tended to increase at higher HSC levels. The control showed a b* value of 18.87, while the 15% and 20% HSC formulations showed higher values of 20.31 and 20.53, respectively (*p* < 0.05). Therefore, HSC incorporation, particularly at higher levels, shifted the color profile toward a slightly less red and more yellow appearance.

The total color difference values ranged from 0.92 to 2.38 among the HSC-containing formulations. The 10% HSC formulation showed the lowest ΔE value of 0.92, indicating the smallest overall color difference relative to the control, whereas the 20% HSC formulation showed the highest ΔE value of 2.38. The 5% and 15% HSC formulations showed intermediate values of 2.13 and 1.59, respectively. These results further confirm that the effect of HSC on color was not strictly concentration-dependent, although the highest incorporation level produced the greatest overall color difference.

The observed color changes may be attributed to the intrinsic pigments and compositional characteristics of HSC, as well as their interactions with the protein–starch matrix during extrusion. Hemp-derived ingredients contain naturally occurring colored compounds that may influence the lightness, redness, and yellowness of the final product. In addition, changes in protein, fiber, and residual lipid contents may affect the extent of heat-induced browning and pigment distribution during HME. Similar changes in color have been reported in high-moisture meat analogs containing hemp-derived ingredients [32], as well as in other hemp-fortified food matrices such as energy bars [33]. Overall, HSC incorporation altered the color profile of the HMMA, but the magnitude and direction of these changes varied depending on the incorporation level.

### 2.6. Texture Properties

#### 2.6.1. Hardness (HD), Springiness (SP), Chewiness (CHE) and Cohesiveness (COH)

The texture properties of the HMMA containing different levels of HSC powder are presented in Table 3. Texture profile parameters are important indicators of the mechanical characteristics, structural integrity, and meat-like textural properties of plant-based meat analogs [34]. Both the first and second hardness values decreased significantly when the HSC incorporation level reached 15% and 20% (*p* < 0.05). HD1 decreased from 44.02 N in the control to 38.88 N at both 15% and 20% HSC, while HD2 decreased from 37.02 N to approximately 32.5 N. Chewiness showed a similar trend, decreasing from 1730.20 g in the control to 1507.38 and 1517.31 g at 15% and 20% HSC, respectively (*p* < 0.05). These results indicate that higher HSC incorporation produced a softer and less chewy HMMA structure. The reductions in hardness and chewiness may be associated with the residual lipids present in HSC. Lipids can exert lubricating and plasticizing effects within the extrusion matrix, reducing intermolecular friction and limiting the development of a highly rigid protein structure. Comparable results have been reported for HMMA containing sunflower meal, in which increasing the level of the oil-containing by-product decreased hardness because of the lubricating effect of its residual lipids [35]. In the present study, the simultaneous reduction in HD1, HD2, and CHE at HSC levels of 15% and 20% suggests that the residual lipid fraction, together with the replacement of refined soy protein and wheat gluten, reduced the rigidity of the extruded matrix.

Despite the decreases in hardness and chewiness, springiness and the hardness degradation ratio remained unchanged across the formulations. Springiness values ranged narrowly from 83.25% to 84.22%, while the hardness degradation ratio remained between 1.19 and 1.20. Cohesiveness also showed only limited variation, ranging from 45.24% to 46.41%, although a significant difference was observed between the 5% and 15% HSC formulations. These results indicate that HSC incorporation primarily affected the force required to deform the HMMA rather than its ability to recover after compression or maintain internal structural continuity. The incorporation of fiber-rich by-products into HMMA formulations can disrupt protein hydration, promote phase separation, and weaken matrix continuity, thereby reducing springiness and cohesiveness [23,36]. In contrast, the relatively stable springiness, cohesiveness, and hardness degradation ratio observed in the present study suggest that HSC incorporation did not substantially disrupt the elastic recovery or internal binding characteristics of the HMMA matrix. Springiness represents the ability of the extrudate to recover its original shape after deformation, whereas cohesiveness reflects the internal bonding strength and resistance of the structure to repeated compression [37,38]. Therefore, the preservation of these parameters indicates that the softer texture produced at higher HSC levels was not accompanied by a pronounced loss of structural integrity.

These textural results are consistent with the thermal–rheological behavior of the formulations. Although the initial storage and loss moduli decreased with increasing HSC content, the final moduli after cooling remained within a range comparable to that of the control formulation (Figure 1). The maintenance of springiness, cohesiveness, and hardness degradation ratio similarly indicates that the HSC-containing formulations retained their capacity to develop a continuous viscoelastic structure during processing. Collectively, the results suggest that HSC incorporation at levels of up to 20% reduced matrix rigidity and chewiness while preserving the elastic recovery and internal structural stability of the HMMA.

#### 2.6.2. Cutting Strength (CST) and Texturization Degree (TD)

Cutting strength (CST) and texturization degree (TD) are commonly used to evaluate the mechanical anisotropy and fibrous characteristics of high-moisture meat analogs. The cutting strengths measured parallel (CSTp) and vertical (CSTv) to the fibrous orientation, together with the calculated TD values, are presented in Table 3. Both CSTp and CSTv generally increased at higher HSC incorporation levels. CSTp increased from 8.32 g/mm^2^ in the control to 9.31 and 9.61 g/mm^2^ at 15% and 20% HSC, respectively (*p* < 0.05). Similarly, CSTv increased from 8.67 g/mm^2^ in the control to 10.34 and 11.03 g/mm^2^ at 15% and 20% HSC, respectively (*p* < 0.05). The 15% and 20% HSC formulations showed significantly higher cutting strengths than the control in both directions. These results indicate that higher HSC incorporation increased the resistance of the extrudates to cutting, despite the concurrent reductions in hardness and chewiness.

Although hardness decreased at HSC incorporation levels of 15% and 20%, the characteristic fibrous structure of the HMMA was maintained, as supported by the cutting-strength results and the macroscopic appearances shown in Figure 2. This difference between compression and cutting behavior suggests that HSC produced a softer bulk texture without weakening the oriented internal structure of the extrudates. Hardness obtained from double-compression testing reflects the overall force required to deform the sample, whereas directional cutting strength is more closely associated with resistance along and across the fibrous structure. Accordingly, the reduction in hardness did not necessarily indicate deterioration of the anisotropic structure.

TD represents the ratio of cutting strength measured in the vertical direction to that measured in the parallel direction and is used as an indicator of structural anisotropy in meat analogs [39]. The TD values remained constant at 1.04 from 0% to 10% HSC and then numerically increased to 1.11 and 1.15 at 15% and 20% HSC, respectively (*p* > 0.05). These results indicate that HSC incorporation did not impair the directional organization of the extrudates. The higher numerical TD values at 15% and 20% HSC were also consistent with the relatively compact and oriented fibrous regions observed in the corresponding samples.

The increases in cutting strength at higher HSC levels may be associated with the residual lipids and proteins present in HSC. Previous studies have shown that moderate lipid levels can influence protein restructuring and aggregation during extrusion cooking [40,41]. Lipids can function as lubricants and plasticizers, modifying melt flow, molecular mobility, and the balance between covalent and non-covalent interactions within the protein matrix [42]. These effects may facilitate structural rearrangement and alignment during passage through the cooling die, while also reducing the rigidity of the overall matrix. This interpretation is consistent with the simultaneous decrease in compression hardness and increase in directional cutting strength observed at 15% and 20% HSC.

The effects of lipids on texturization depend strongly on their concentration and interactions with other formulation components. Excessive lipid levels may reduce barrel friction and mechanical energy transfer, thereby weakening protein texturization [34,42,43,44]. In the present formulations, however, the residual lipids introduced through HSC did not disrupt fibrous structure formation. Instead, the higher cutting strengths and maintained TD values suggest that the combined protein, fiber, and lipid fractions of HSC supported the formation of a softer but structurally oriented HMMA matrix. Overall, incorporation of up to 20% HSC maintained the anisotropic fibrous characteristics of the extrudates while increasing their resistance to directional cutting.

## 3. Conclusions

This study investigated the effects of HSC incorporation on the physicochemical, thermal–rheological, textural, and structural properties of HMMA produced by HME. The results demonstrated that HSC can be incorporated up to 20% while maintaining the essential quality and fibrous structure of the extrudates, despite minor modifications in pasting behavior, texture, cutting strength, and color.

Increasing the HSC level progressively reduced peak viscosity, indicating changes in the hydration and viscosity development of the protein–starch matrix. Crucially, regarding the gel properties, all formulations consistently exhibited an elastic-dominant gel behavior (G′ > G″) across the entire thermal processing cycle. Although the initial G′ and G″ values tended to decrease at higher HSC levels, all formulations exhibited G′ values greater than G″ throughout the temperature cycle, and the viscoelastic moduli increased substantially during heating and cooling. The final moduli of the HSC-containing blends remained comparable to those of the control. These findings indicate that HSC altered the initial viscoelastic characteristics of the formulations while preserving their capacity to develop a reinforced gel-like network during thermal treatment. From a textural perspective, incorporation of 15% and 20% HSC significantly reduced hardness and chewiness, resulting in a softer HMMA structure. In contrast, springiness, cohesiveness, and the hardness degradation ratio showed only limited changes, indicating that elastic recovery and internal structural continuity were largely maintained. Cutting strength in both the parallel and vertical directions increased at the higher HSC levels, while the texturization degree increased numerically from 1.04 in the control to 1.15 at 20% HSC. Together with the macroscopic appearance of the extrudates, these results demonstrate that HSC incorporation produced a softer bulk texture while maintaining the anisotropic fibrous structure of the HMMA.

Overall, cold-pressed HSC demonstrates strong potential as an upcycled ingredient to partially replace conventional soy protein, wheat gluten, and corn starch in HMMA formulations, maintaining thermal-network-forming capacity and fibrous structural characteristics up to a 20% substitution level. Further studies should clarify the individual contributions of HSC proteins, dietary fiber, and residual lipids to network formation using complementary structural and molecular analyses. Evaluating HSC incorporation levels above 20% together with optimization of extrusion conditions and sensory evaluation would also help define its practical application range in PBMA.

## 4. Materials and Methods

### 4.1. Materials

Isolated soybean protein (ISP; >90% crude protein, 6.8% moisture; Pingdingshan TianJing Plant Albumen Co., Ltd., Pingdingshan, China), wheat gluten (WG; >75% crude protein, <8.0% moisture; Roquette Frères, Lestrem, France), and corn starch (CS; 90.0% starch content, 10.1% moisture; Samyang, Ulsan, Republic of Korea) were utilized. HSC, obtained as a cold-pressing residue of hemp seeds from Hemp & R Bio Co., Ltd. (Andong, Republic of Korea), was milled and sieved through a 40 mesh sieve. The proximate composition of HSC consisted of 6.89% moisture, 25.56% crude fat, 31.45% crude protein, 5.80% crude ash, and 30.30% total carbohydrate (calculated by difference).

### 4.2. Pasting Properties of HSC Blends (RVA)

The pasting profiles of the HSC-incorporated blends were measured utilizing a Rapid Visco Analyzer (RVA 4800; Perten Instruments, PerkinElmer, Sydney, NSW, Australia) in accordance with AACC Method 76-21.01 [45], with slight modifications. To achieve a standardized 14% moisture basis, the sample mass was precisely adjusted based on its initial moisture content. To prepare the test suspension, roughly 3.0 g of the sample (as-is basis) was suspended in 25.0 ± 0.1 mL of distilled water in an RVA canister. The temperature profile was initiated by equilibrating the mixture at 50 °C for 1 min, followed by heating to 95 °C at a rate of 12 °C/min. After maintaining the temperature at 95 °C for 2.5 min, the suspension was cooled down to 50 °C at 12 °C/min and held for 2 min. The paddle was initially spun at 960 rpm for 10 s to achieve a homogeneous dispersion, after which the speed was moderated to 160 rpm for the remaining analysis period. Viscosity data were collected at 4 s intervals, and all analyses were measured in triplicate.

### 4.3. Preparation of HSC Blends for Rheological Analysis

Dry blends were prepared according to the formulations listed in Table 4, with HSC powder substituted at 0, 5, 10, 15, and 20% (*w*/*w*). The baseline matrix comprised isolated soy protein (ISP), wheat gluten (WG), and corn starch (CS) blended at a weight ratio of 60:30:10. To conduct rheological characterizations, these dry formulations were dispersed in distilled water to achieve a final concentration of 20% (*w*/*v*) and agitated at 300 rpm for 1 h under ambient temperature. Prior to rheological testing, the resultant suspensions were refrigerated at 4 °C for 24 h to ensure sufficient and thorough hydration.

### 4.4. Thermal–Rheological Properties of HMMA Blends

To evaluate the rheological properties of HMMA mixtures, a rotational rheometer (MCR 302e, Anton Paar Co., Graz, Austria) fitted with a 50 mm stainless steel parallel plate was utilized, modifying a previously reported procedure [27]. Thermal sweep measurements were carried out at 1 Hz frequency and 1% strain (confirmed to be within the linear viscoelastic range) after allowing the sample to rest for 5 min at 25 °C. The system was heated to 95 °C from 25 °C (5 °C/min), maintained for 5 min, and cooled back to 25 °C under identical rate conditions. Approximately 2.5 g of specimen was loaded onto the bottom plate center with a 1 mm testing gap. Moisture loss was minimized by sealing the outer boundaries with silicone oil and attaching a solvent trap.

### 4.5. HMMA Extrusion Process

**Figure 3:**
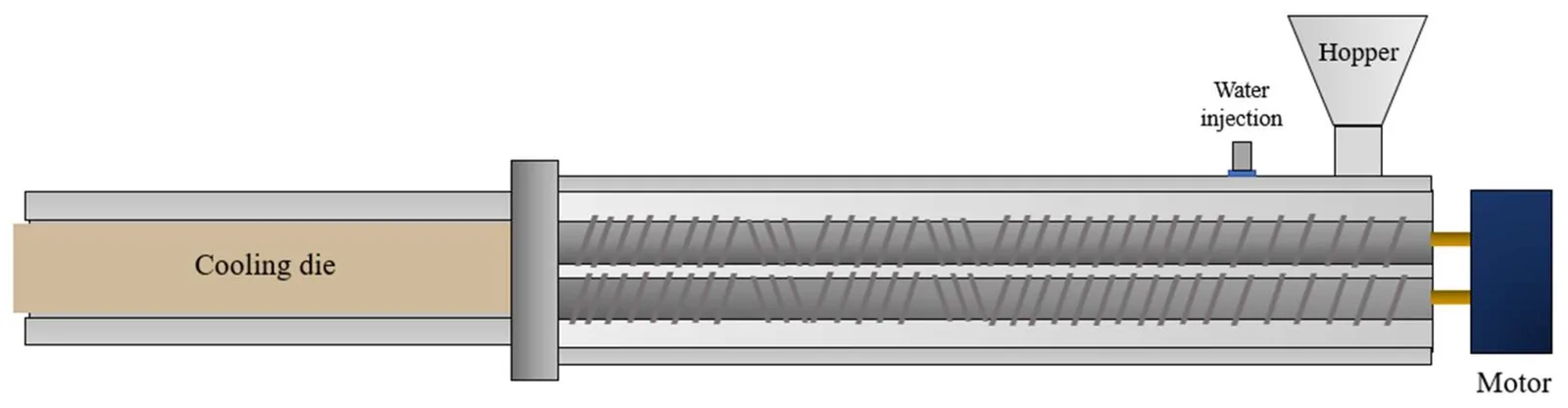
Configuration of the HME setup.

Experiments were conducted on a co-rotating twin-screw extrusion system featuring a temperature-regulated cooling die (Myung Kim Extrusion Co., Daejeon, Republic of Korea) (Figure 3). Screws with a diameter of 25 mm and a 40:1 length-to-diameter (L/D) proportion were utilized in the extruder. The substitution level of HSC was set up to 20% based on preliminary trials to ensure process stability and product quality. The HME process was conducted based on previously established methods with slight modifications [23,36]. Extrusion processing was executed at a screw rotation speed of 300 rpm, a feed moisture content of 65%, and a material throughput of 90 g/min. The temperature across the extruder barrels was regulated to 150 °C. The mean residence time of the material in the extruder barrel was approximately 60–90 s, and extrudates were collected for 15 min after the system reached steady-state processing conditions (stable torque and die pressure). Extrudate samples for subsequent physical and textural analyses were randomly collected at three different time points during the 15-min steady-state production period for each formulation.

### 4.6. Expansion Ratio (ER) and Bulk Density (BD)

To characterize the physical attributes of the HMMA containing HSC, both expansion degree and volumetric mass density were quantified. Cross-sectional profiles (width and height) of the extrudates were recorded via a digital vernier caliper (942000 IP67, Brütsch/Rüegger Tools Ltd., Urdorf, Switzerland); these values were normalized against the exit die dimensions to establish the expansion ratio according to Equation (1). For density assessment, an analytical balance (BA310, DAIHAN Co., Ltd., Wonju, Republic of Korea) was employed to weigh the samples. Sample volumes were subsequently derived from 3D geometry measurements (width, depth, and height), enabling the calculation of bulk density as outlined in Equation (2).

(1) ER = A_extudate_/A_die_

where A_extrudate_ is the cross-sectional area of the extrudate and A_die_ is the cross-sectional area of the extrusion die.

(2) BD (g/cm^3^) = Mass of the extrudate/Volume of the extrudate

### 4.7. Color Measurement

To evaluate the chromatic profiles of HMMA, samples were freeze-dried and ground into powder prior to analysis using a spectrophotometer (CM-5, Minolta Co., Ltd., Osaka, Japan). The instrument was calibrated prior to measurement using the standard white and black calibration plates according to the manufacturer’s instructions. Tristimulus color coordinates lightness (L*), redness (a*), and yellowness (b*) were measured in triplicate, with the average values utilized for data interpretation. The total color variance (∆E) was computed as described in Equation (3).

(3) ΔE = {(L* − L)^2^ + (a* − a)^2^ + (b* − b)^2^}^1/2^

### 4.8. Texture Profile Analysis (TPA) and Cutting Strength (CS)

The texture profile analysis and cutting strength of the meat analogs were determined using a texture analyzer (Z0.5 TS, Zwick Roell, Ulm, Germany) to digitally characterize the texture features. Cubic specimens (1 cm × 1 cm) underwent double-compression testing using a 100 mm cylindrical probe up to a peak force of 600 N, with six replicates per treatment. The first hardness (HD_1_), second hardness (HD_2_), hardness degradation ratio (HDR), springiness (SP), cohesiveness (COH), and chewiness (CHE) were calculated using Equations (4)–(7), as modified from Trinh and Glasgow [37]. Cutting strength (CS) was evaluated by applying a 70 mm × 3 mm probe in both parallel and vertical directions relative to the fiber direction at a 600 N maximum loading capacity (n = 6). The shear force values were calculated using Equation (8), and the extent of fibrous structure was evaluated according to Equation (9).

(4) HDR = HD_1_/HD_2_

where HD_1_ is the maximum force of load application in the first cycle and HD_2_ is the maximum force of load application in the second cycle.

(5) SP (%) = D_2_/D_1_ × 100

where D_1_ represents the compression distance applied during the first cycle, and D_2_ corresponds to the recovered height measured during the second compression.

(6) COH (%) = A_2_/A_1_ × 100

where A_1_ reflects the work area generated during the first compression cycle, and A_2_ denotes the work area measured during the second compression.

(7) CHE (g) = Springiness × Cohesiveness × Hardness

(8) CST (g/mm^2^) = Maximum stress/cross sectional area

(9) Texturization degree of cutting strength (TD) = CST_v_/CST_p_

where CST_v_ is the cutting strength in the vertical direction and CST_p_ is the parallel direction.

### 4.9. Statistical Analysis

Statistical processing of all experimental results was performed using IBM SPSS Statistics (Version 23.0, IBM Corp., Armonk, NY, USA). Data are expressed as mean ± standard deviation. The number of replicates for each analysis is specified in the corresponding methodological subsection. Normality and homogeneity of variance were assessed prior to one-way ANOVA. Tukey’s HSD test was used for post-hoc comparisons at *p* < 0.05.

## Figures and Tables

**Table 1:** Paste viscosity of HMMA blends at different levels of HSC powder content.

HSC Powder (%)	Paste Viscosity (cP)					PT (°C)	Peak Time (min)
	PV ^(^^1)^	TV	BV	FV	SB		
0	2098.33 ± 97.50 ^a^	1658.67 ± 26.95 ^a^	439.67 ± 80.45 ^ab^	4089.00 ± 133.91 ^a^	2430.33 ± 116.66 ^a^	50.00 ± 0.00 ^a^	7.64 ± 0.08 ^b^
5	1801.00 ± 81.46 ^b^	1128.00 ± 50.27 ^b^	673.00 ± 99.23 ^a^	3428.00 ± 94.62 ^b^	2300.00 ± 69.09 ^a^	50.00 ± 0.00 ^a^	8.31 ± 0.10 ^b^
10	1621.00 ± 176.69 ^bc^	1109.33 ± 44.06 ^b^	511.67 ± 220.73 ^a^	3436.33 ± 169.67 ^b^	2327.00 ± 143.63 ^a^	50.00 ± 0.00 ^a^	10.40 ± 1.43 ^a^
15	1353.67 ± 54.64 ^cd^	1157.00 ± 42.51 ^b^	196.67 ± 58.53 ^b^	3071.33 ± 140.22 ^c^	1914.33 ± 134.66 ^b^	50.00 ± 0.00 ^a^	11.67 ± 0.70 ^a^
20	1257.67 ± 17.01 ^d^	1062.33 ± 20.01 ^b^	195.33 ± 13.50 ^b^	2791.00 ± 47.09 ^c^	1728.67 ± 35.70 ^b^	50.00 ± 0.00 ^a^	11.64 ± 0.20 ^a^

^(1)^ PV (peak viscosity), TV (trough viscosity), BV (breakdown), FV (final viscosity), SB (setback), and PT (pasting temperature). Values with different letters in the same column indicate significant differences (*p* < 0.05) by Tukey’s HSD test.

**Table 2:** Color properties, expansion ratios and bulk densities of HMMA at different levels of HSC powder content.

HSC Powder (%)	Color Value			ΔE	ER	BD (g/cm^3^)
	L*	a*	b*			
0	76.52 ± 0.31 ^b^^(^^1)^	2.59 ± 0.10 ^a^	18.87 ± 0.90 ^b^	-	1.17 ± 0.03 ^bc^	0.99 ± 0.03 ^a^
5	74.92 ± 0.08 ^c^	2.68 ± 0.04 ^a^	20.28 ± 0.20 ^ab^	2.13	1.19 ± 0.02 ^b^	0.97 ± 0.02 ^a^
10	77.27 ± 0.13 ^a^	2.08 ± 0.04 ^b^	19.01 ± 0.13 ^b^	0.92	1.20 ± 0.03 ^b^	0.99 ± 0.03 ^a^
15	76.20 ± 0.10 ^b^	1.99 ± 0.02 ^b^	20.31 ± 0.29 ^ab^	1.59	1.16 ± 0.04 ^c^	1.00 ± 0.04 ^a^
20	74.90 ± 0.17 ^c^	2.06 ± 0.04 ^b^	20.53 ± 0.14 ^a^	2.38	1.24 ± 0.03 ^a^	0.91 ± 0.03 ^b^

^(1)^ Values with different letters in the same column indicate significant differences (*p* < 0.05) by Tukey’s HSD test. ER (expansion ratio), BD (bulk density).

**Table 3:** Texture properties, cutting strength and texturization degree of HMMA at different levels of HSC powder content.

HSC Powder (%)	Hardness (N)		HDR	SP (%)	CHE (g)	COH (%)	CST (g/mm^2^)		TD
	HD_1_^(^^1)^	HD_2_					CST_p_	CST_v_	
0	44.02 ± 0.96 ^a^	37.02 ± 0.80 ^a^	1.19 ± 0.00 ^a^	83.62 ± 0.67 ^a^	1730.20 ± 38.16 ^a^	46.12 ± 0.28 ^ab^	8.32 ± 0.25 ^b^	8.67 ± 0.23 ^b^	1.04 ± 0.04 ^a^
5	43.38 ± 0.86 ^a^	36.07 ± 0.63 ^ab^	1.20 ± 0.01 ^a^	83.58 ± 0.87 ^a^	1715.65 ± 47.09 ^a^	46.41 ± 1.01 ^a^	7.48 ± 0.37 ^c^	7.76 ± 0.78 ^b^	1.04 ± 0.14 ^a^
10	42.67 ± 0.43 ^a^	35.57 ± 0.44 ^b^	1.20 ± 0.01 ^a^	84.22 ± 0.42 ^a^	1665.16 ± 26.68 ^a^	45.43 ± 0.56 ^ab^	8.06 ± 0.50 ^bc^	8.31 ± 0.15 ^b^	1.04 ± 0.09 ^a^
15	38.88 ± 1.10 ^b^	32.48 ± 0.92 ^c^	1.20 ± 0.00 ^a^	84.02 ± 0.27 ^a^	1507.38 ± 61.74 ^b^	45.24 ± 0.78 ^b^	9.31 ± 0.33 ^a^	10.34 ± 0.62 ^a^	1.11 ± 0.05 ^a^
20	38.88 ± 1.30 ^b^	32.50 ± 1.11 ^c^	1.20 ± 0.01 ^a^	83.25 ± 1.12 ^a^	1517.31 ± 35.82 ^b^	45.97 ± 0.53 ^ab^	9.61 ± 0.60 ^a^	11.03 ± 0.87 ^a^	1.15 ± 0.07 ^a^

^(1)^ HD_1_ (first hardness), HD_2_ (second hardness), HDR (hardness degradation ratio), SP (springiness), CHE (chewiness), COH (cohesiveness), CST (cutting strength), CST_p_ (cutting strength in the parallel direction), CST_v_ (cutting strength in the vertical direction), TD (texturization degree). Values with different letters in the same column indicate significant differences (*p* < 0.05) by Tukey’s HSD test.

**Table 4:** Formulation of HMMA blends at different levels of HSC powder content.

HSC ^(^^1)^ (%)	ISP (%)	WG (%)	CS (%)
0	60	30	10
5	57	28.5	9.5
10	54	27	9
15	51	25.5	8.5
20	48	24	8

^(1)^ HSC (hemp seed cake), ISP (isolated soy protein), WG (wheat gluten), CS (corn starch).

## Data Availability

The original contributions presented in this study are included in the article. Further inquiries can be directed to the corresponding author.
